# Comparison of the OHIP-14 and GOHAI as measures of oral health among elderly in Lebanon

**DOI:** 10.1186/1477-7525-10-131

**Published:** 2012-10-30

**Authors:** Nada El Osta, Stephanie Tubert-Jeannin, Martine Hennequin, Nada Bou Abboud Naaman, Lana El Osta, Negib Geahchan

**Affiliations:** 1 Department of Prosthetic Dentistry, School of Dentistry, Saint-Joseph University, Beirut, Lebanon; 2 Department of Public Health, School of Medicine, Saint-Joseph University, Beirut, Lebanon; 3 Clermont University, University of Auvergne, Centre de Recherche en Odontologie Clinique-EA4847, BP-10448, F-63000 Clermont-Ferrand, France; 4 CHU of Clermont-Ferrand, Department of odontology, Hôtel-Dieu, F-63000 Clermont-Ferrand, France; 5 Department of Periodontology, School of Dentistry, Saint-Joseph University, Beirut, Lebanon

**Keywords:** Oral health, Quality of Life, Lebanese elderly, Psychometric properties, Health status indicators, Dental health survey

## Abstract

**Background:**

The respective abilities of the GOHAI and OHIP-14 to discriminate between aged patients with different levels of oral diseases have rarely been studied in developing countries. The aim of this study was to compare the discriminative abilities of the OHIP-14 and the GOHAI in an elderly Lebanese population, and particularly to identify persons with different masticatory function.

**Methods:**

A sample of elderly, aged 65 years or more, living independently was recruited in two primary care offices in Beirut, Lebanon. Data were collected by means of personal interview and clinical examination. The Arabic OHIP-14 and GOHAI questionnaires were used after cultural adaptation for use in Lebanon. The internal consistency, reproducibility and concurrent validity were verified. To test their discriminative abilities, the ADD (GOHAI and OHIP) and SC (GOHAI and OHIP) scores were dichotomized according to the 25^th^ and 75^th^ percentile respectively and logistic regressions were conducted using socio-demographic, clinical and subjective explanatory variables.

**Results:**

Two hundred and six participants were included; mean age was 72 years and 60% were women. Good psychometric properties were observed for both questionnaires for internal consistency (Cronbach’s alpha>0.88), reproducibility (ICC>0.86) and concurrent validity. Strong correlations were found between GOHAI and OHIP-14 scores but a high prevalence of subjects with no impact was observed using the OHIP-14. Both questionnaires were able to discriminate between participants according to age, perception of temporomandibular joint (TMJ) pain or functional status as represented by the number of dental Functional Units (FU). GOHAI was more discriminant since it identified participants with high dental care needs: high numbers of decayed teeth, low numbers of teeth and socially deprived status.

**Conclusions:**

Lebanese elderly with high dental care needs and impaired oral health were identified more easily with the GOHAI. These results may guide the choice of dental indicators to use in a national geriatric survey.

## Background

In some developing countries, the aged population is expanding; in Lebanon, local statistical reports indicate that individuals aged 65 years and over make up around 10% of the total population [1]. The oral health of the Lebanese elderly population has never been evaluated. Lebanon is a country characterized by a free economy with no generalized public health insurance system. The dental care system is therefore not accessible for older people with low incomes [2]. As in other countries, it can be hypothesized that high levels of oral disease in Lebanese elderly would be associated with an impaired quality of life. Elderly who have lost many teeth and cannot afford the cost for dentures may have important functional limitations and consequent nutritional problems. Moreover, the presence of carious teeth may lead to infection, pain and discomfort [3].

Investigators wishing to evaluate oral health of Lebanese elderly face the problem of selecting the most appropriate oral indicators. A variety of Oral Health Related Quality of Life (OHRQoL) instruments have been developed to evaluate the functional and psychosocial impacts of oral diseases. The main objective of OHRQoL questionnaires has rarely been clearly defined. Some measures of OHRQoL as of Health Related Quality of Life (HRQoL) may be intended to distinguish different clinical status (discriminative instrument) while others could be intended to evaluate within-subject changes with time (sensitivity to change) [4,5]. According to those characteristics, the questionnaires may be used for different circumstances or objectives; in clinical trials to detect differences in treatment effect and in epidemiological surveys to measure the health of populations and to provide information for policy decisions [6].

Among available OHRQoL instruments, the Geriatric Oral Health Assessment Index (GOHAI) and the Oral Health Impact Profile (OHIP-14) have been validated, firstly, in elderly populations. They were initially developed in English-speaking countries (USA, Australia) [7-10], and were translated and validated for use in several countries [11-16]. Recently, GOHAI and OHIP-49 have been translated into Arabic and validated for use in Jordan and Saudi Arabia [17-19]. In order to choose a suitable OHRQoL instrument, there is a need to consider its discriminative performances for measuring the oral health of a population [7]. The ability of OHRQoL questionnaires to evaluate oral health may also vary depending on the type of population. Some comparisons of the properties of the OHIP-14 and the GOHAI have already been performed among elderly in Canada, Germany and Japan [20-23]. Results indicate that the distributions of the two measures were different, with a high proportion of subjects with no impact when using the OHIP-14. Moreover, it was shown that the GOHAI and OHIP questionnaires have slightly different discriminative capacities depending of the type of variables considered [20]. The GOHAI seem to be more strongly related to masticatory performances whereas OHIP could be a better predictor of depression [21-23]. No comparison study has been done in Arabic countries where the cultural context and the dental care system are different. Moreover, the respective abilities of the GOHAI and OHIP-14 to discriminate between aged patients with different levels of oral disease and different dental functional status have rarely been studied.

The aim of this study was to compare the psychometric properties and discriminative abilities of the OHIP-14 and the GOHAI in an elderly population in Lebanon and to particularly to explore the ability of these instruments to distinguish between patients with different functional status measured by the number of dental functional units (FU).

## Methods

### The GOHAI and OHIP-14 questionnaires

The GOHAI is intended to report oral function problems and psychosocial impacts associated with oral diseases. The 12 items of the GOHAI assess three dimensions which are physical function, pain & discomfort and psychosocial function [8]. The OHIP assesses the social impacts of oral disorders. The questionnaire evaluates dysfunction, discomfort and disability caused by oral disorders. The 14 items of the OHIP-14 incorporate seven dimensions relating to functional limitation, physical pain, psychological discomfort, physical disability, psychological disability, social disability and handicap [10,24-26].

Subjects are asked if they have always/very often, often, sometimes, seldom or never experienced any of those problems in the past three months. Responses are scored on a scale ranging from 1 to 5. The summary scores range from 12 to 60 for the ADD-GOHAI and from 14 to 70 for the ADD-OHIP-14 with a higher score indicating better oral health. The simple count scores (SC-GOHAI or SC-OHIP-14) are obtained by counting the number of items with responses ‘sometimes’, ‘often’ or ‘always/very often’. Using this approach, scores range from 0 to 12 and 0 to 14 for the SC-GOHAI and the SC-OHIP-14 respectively with a higher score indicating a poorer oral health.

### Adaptation of the OHIP-14 and GOHAI for use in Lebanon

The validated Arabic versions of the OHIP-14 and GOHAI questionnaires were available [17,18] but a cultural adaptation was necessary for use in Lebanon [27]. The questionnaires were discussed with experts in the field of geriatrics, gerodontology and Arabic language and no modification was required. A pilot study was conducted in a sample of Lebanese elderly (n=87) to ensure that the previously validated Arabic versions of GOHAI and OHIP-14 were suitable for use in Lebanon. The meaning, comprehensibility and acceptability of the OHIP-14 and GOHAI questions were studied by means of individual interviews because parts of the subjects were illiterate. In the GOHAI questionnaire, patients answered the negatively worded questions more easily and more appropriately [28]. Three items initially worded positively (items 3, 5, 7) were therefore modified and re-worded negatively. A back translation was conducted for the three modified items in order to ensure their linguistic equivalence. For the OHIP-14 questionnaire, the answer ‘very often’ was replaced by ‘always’ because people were not able to distinguish the words ‘often’ and ‘very often’. The adapted versions of the two questionnaires were pilot tested again in order to ensure their comprehensibility.

### Study population

Participants were recruited in two different primary health care offices in Beirut, Lebanon: a dispensary where persons with no health insurance can have free medical consultations and a private office where patients pay for medical fees or are covered by a health insurance. During a three-month period (mid July to mid October 2011) and 2 days a week, all patients aged 65 years or more were invited to participate in the study. In order to guarantee that only individuals able to give informed consent were recruited, the medical staff has reviewed patients’ medical charts and excluded patients with cognitive, neurological, psychiatric disorders or with acute systemic disease (n=7). Accessibility to the elderly population is difficult especially in Lebanon where there is no social security or national health system covering the entire population. Patients were thus recruited in the area of Beirut that concentrates the highest percentage of Lebanese elderly (13.6%) compared to other region in Lebanon [1,29]. Furthermore, two different health facilities were chosen in order to allow the recruitment of patients with varied socio-demographic profiles. The number of 200 subjects to be included was arbitrarily set, taking into account the sample sizes used in previous similar studies [20-23].

The protocol for this study was submitted to the ethical research committee at Saint-Joseph University of Beirut, Lebanon (Ref: CEE 366). Written informed consent was obtained from the participants.

### Data collection

Data were collected from questionnaires administered by an interviewer and from a clinical oral examination. In addition to the GOHAI and OHIP-14 items, the questionnaire included socio-demographic data such as age, gender, educational level and recruitment setting. Participants were also asked about their perception of their general and oral health status, whether they were satisfied with their dental conditions and their feelings about their need for dental treatment. They were also asked if they were currently receiving dental treatment and whether they suffered from dry mouth or temporomandibular joint (TMJ) pain.

The same examiner performed all oral examinations, the same day as the questionnaire was administered in medical examination rooms. Portable lamps, equipment and pre-packaged sterilized instruments were used. The examinations, based on 28 teeth, used World Health Organization criteria to register decayed (D_3_ level, for coronary and root lesions), missing and filled teeth [30]. The presence and types of dentures used by the participants were also recorded. Finally, the number of dental functional units (FU) was evaluated by recording the number of tooth pairs participating in mastication. The FU number was evaluated by asking the subjects to chew 1–2 cycles on 200 μm thick articulating paper; the number of teeth in the mandibular arch that had at least one coloured mark gave the number of FUs. Patients with dentures were asked if they had used their denture during recent meals. The number of FUs was recorded without the denture for those who had not used their denture during recent meals [31,32].

### Data analysis

The statistical analysis was performed using a software program (SPSS for Windows version 17.0, USA). The alpha error was set at 0.05. Reliability was assessed by examining internal consistency and reproducibility. Spearman’s rank correlation coefficients were used to measure inter-item and item-score correlations. Cronbach's alpha and alpha, if an item was deleted, were calculated to assess the degree of internal consistency. Reproducibility was assessed by repeating the administration of the GOHAI and OHIP-14 to 31 subjects three weeks after the first administration. Stability was measured by using intra-class correlation coefficients (ICC) for the different global scores and for each item calculated with a two-way random effects model.

Since there is no gold standard for OHRQoL indices, the validation process relies on the evaluation of concurrent validity, which examines a logical hypothesis by testing the index against a proxy measure of a similar concept. It was hypothesized that subjects with lower OHRQoL ADD-scores or higher-SC scores would be less satisfied with their mouths, and would report higher self-rated treatment need and pain in the last three months, and would have poorer self-rated oral and general health. As the scores derived from the GOHAI and OHIP-14 were not normally distributed, Mann–Whitney and Kruskal-Wallis tests were used.

Discriminant validity was evaluated by comparing the GOHAI and OHIP-14 scores between different groups with objectively assessed dental status. It was hypothesized that patients with high levels of oral disease and poor dental status (patients with perception of TMJ pain or dry mouth, under dental treatment, edentulous without dentures, with a high number decayed teeth (≥7), with a low number of teeth (<22) or FUs (≤4) would have lower ADD-scores and higher SC-scores. The cut-off values for continuous clinical variables were chosen using the 50^th^ and 75^th^ percentiles. It was also hypothesized that GOHAI and OHIP-14 scores could discriminate between participants with different socio-demographic characteristics such as age, gender, level of education and recruitment setting.

To test the discriminative properties of OHIP and GOHAI scores, the ADD (GOHAI and OHIP) and SC (GOHAI and OHIP) scores were dichotomized using the 25^th^ and 75^th^ percentile respectively (Table 1). This allowed the ability of the OHRQoL questionnaires to identify patients with high dental needs to be evaluated. Cross tabulations were performed and Odds Ratios (ORs) calculated. Four logistic regression models were carried out with one categorical OHRQoL dependent variable and explanatory independent variables. Explanatory variables that were not related to GOHAI or OHIP scores in the univariate analysis with p-values >0.25 were not included in the logistic regressions.

**Table 1:** Descriptive statistics for GOHAI and OHIP-14 scores

	**ADD-GOHAI**	**SC-GOHAI**	**ADD-OHIP-14**	**SC-OHIP-14**
**Range**	13-60	0-12	20-70	0-14
**Mean ±SD**	46.7±11.2	4.2±3.4	62.1±9.3	2.8±3.3
**Median**	48.5	3	65.0	2
**25**^**th **^**percentile**	39.75	1.00	58.00	.00
**75**^**th **^**percentile**	56.00	7.00	69.25	4.00
**Absence of impact (%)**	8.7%	15.5%	24.8%	33.5%

## Results

### Characteristics of the participants

Two hundred and six participants were recruited from the primary care offices: 122 in the dispensary and 84 in the private medical structure. All participants answered the interviewer-administered questionnaires and were clinically examined. The majority of participants were women (60%). The mean age was 72 years (± 6.35). Forty-six (22.3%) participants reported having completed high school education while others had stopped their studies earlier.

In the present sample, the proportion of women was high compared to the whole Lebanese elderly population (49.6%). The percentage of illiterate people in the sample (21.95%) was low compared to that of the Lebanese elderly population (41.4%) but higher than the one observed for the population (all ages) living in Beirut (4.1%). On the contrary, the proportion of patients who had graduated high school was low compared to the population (all ages) of Beirut (38%) but higher than the one observed in elderly in Lebanon (9.8%) [29].

The majority (63%) were dentate and among them, 34% wore denture(s). Only 78% of the edentulous patients had upper and lower prostheses. The mean number of missing teeth, decayed teeth and number of FUs were respectively 17.8 (± 9.90), 3.32 (±4.64) and 4.70 (±3.08). Participants reported having sensations of dry mouth (68.4%) and TMJ pain (16.5%) during the last three months. Approximately one person in three (31.6%) reported fair/poor oral health and 34.5% were dissatisfied or very dissatisfied with their oral health. Moreover, 56.8% reported needs for dental treatment, 5.34% were under dental care and 25.7% reported feeling they were in poor health.

### Distribution of GOHAI and OHIP-14

**Figure 1:**
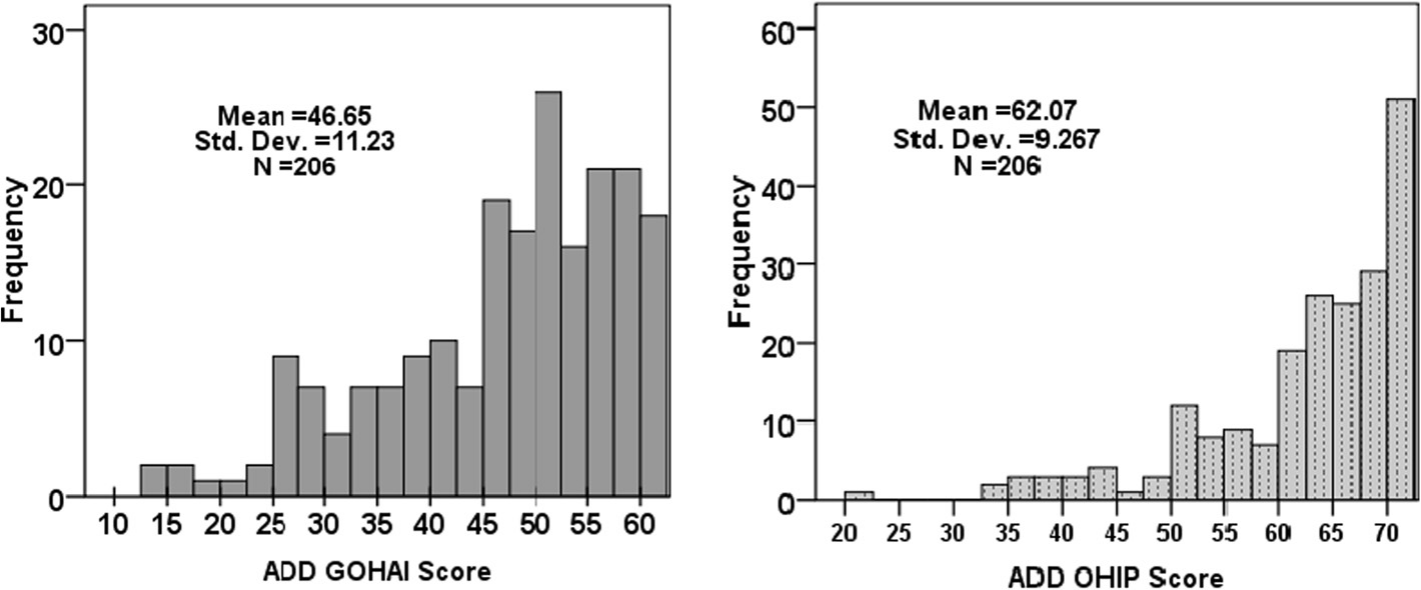
Distribution of ADD-GOHAI and ADD-OHIP scores.

The distributions of the GOHAI and OHIP-14 additive scores are presented Figure 1. The OHIP-14 scores were much more highly skewed than the GOHAI scores. The median value (48.5) for ADD-GOHAI was much lower than the value (65) observed for the ADD OHIP-14 score. The proportion of subjects with no impact varied greatly, from 15.5% for SC-GOHAI and 33.5% for SC-OHIP-14 (Table 1). Nevertheless, the correlation between GOHAI and OHIP-14 scores was high and similar for ADD scores (0.889) and SC scores (0.895).

The responses to the different questions of the GOHAI and OHIP-14 questionnaires are listed Tables 2 and 3. Within the GOHAI questionnaire, oral impacts were frequent for item 2: 41.3% of the participants reported ‘always’ having difficulties when chewing. On the other hand, a small number of participants (4.9%) used medications ‘often or always’ to relieve dental pain (item 8). Within the OHIP-14 questionnaire, oral impacts were frequently reported for question 4; 57.3% of the participants were uncomfortable (seldom to always) when eating. Severe impacts, such as ‘difficulty doing job’ (item 12) or ‘totally unable to function’ (item 14), were mentioned by fewer than 7% of the participants.

**Table 2:** Frequency distribution of the responses for GOHAI items

**Items**	**In the past three months**	**5**	**4**	**3**	**2**	**1**
		**Never**	**Seldom**	**Sometimes**	**Often**	**Always**
	**Physical function**					
1	Limit the kind of food	77 (37.4%)	12 (5.8%)	38 (18.4%)	7 (3.4%)	72 (35%)
2	Trouble biting/chewing	53 (25.7%)	13 (6.3%)	37 (18%)	18 (8.7%)	85 (41.3%)
3	Trouble swallowing	135 (65.5%)	16 (7.8%)	36 (17.5%)	7 (3.4%)	12 (5.8%)
4	Unable to speak clearly	142 (68.9%)	9 (4.4%)	35 (17%)	5 (2.4%)	15 (7.3%)
	**Pain and Discomfort**					
5	Discomfort when eating	88 (42.7%)	31 (15.0%)	60 (29.1%)	12 (5.8%)	15(7.3%)
8	Medications for pain	144 (69.9%)	17 (8.3%)	35 (17%)	1 (0.5%)	9(4.4%)
12	Sensitive teeth	144 (69.9%)	15 (7.3%)	20 (9.7%)	4 (1.9%)	23(11.2%)
	**Psychosocial impacts**					
6	Limit contacts with others	164 (79.6%)	11 (5.3%)	18 (8.7%)	1 (0.5%)	12 (5.8%)
7	Unhappy with appearance	101 (49.0%)	9 (4.4%)	31 (15.0%)	10 (4.9%)	55 (26.7%)
9	Worried or concerned	117 (56.8%)	24 (11.7%)	33 (16.0%)	8 (3.9%)	24 (11.7%)
10	Nervous, self-conscious	129 (62.6%)	10 (4.9%)	32 (15.5%)	9 (4.4%)	26 (12.6%)
11	Uncomfortable eating in front of others	140 (68.0%)	6 (2.9%)	24 (11.7%)	4 (1.9%)	32 (15.5%)

**Table 3:** Frequency distribution of the responses for OHIP items

**Items**	**In the past three months**	**5**	**4**	**3**	**2**	**1**
		**Never**	**Seldom**	**Sometimes**	**Often**	**Always**
	**Functional limitation**					
1	Trouble pronouncing words	140 (68.0%)	11 (5.3%)	33 (16.0%)	6 (2.9%)	16 (7.8%)
2	Sense of taste worse	149 (72.3%)	13 (6.3%)	25 (12.1%)	7 (3.4%)	12 (5.8%)
	**Physical pain**					
3	Painful aching in mouth	123 (59.7%)	21 (10.2%)	53 (25.7%)	4 (1.9%)	5 (2.4%)
4	Uncomfortable to eat	88 (42.7%)	27 (13.1%)	57 (27.7%)	17 (8.3%)	17 (8.3%)
	**Psychological discomfort**					
5	Self-conscious	140 (68.0%)	6 (2.9%)	24 (11.7%)	3 (1.5%)	33 (16.0%)
6	Felt tense	136 (66.0%)	23 (11.2%)	35 (17.0%)	7 (3.4%)	5 (2.4%)
	**Physical disability**					
7	Unsatisfactory Diet	119 (57.8%)	14 (6.8%)	30 (14.6%)	19 (9.2%)	24 (11.7%)
8	Had to interrupt meals	163 (79.1%)	15 (7.3%)	19 (9.2%)	7 (3.4%)	2 (1.0%)
	**Psychological disability**					
9	Difficult to relax	172 (83.5%)	9 (4.4%)	20 (9.7%)	4 (1.9%)	1 (0.5%)
10	Embarrassed	150 (72.8%)	17 (8.3%)	30 (14.6%)	7 (3.4%)	2 (1.0%)
	**Social disability**					
11	Irritability with others	191 (92.7%)	3 (1.5%)	11 (5.3%)	0 (0.0%)	1 (0.5%)
12	Difficulty doing usual jobs	192 (93.2%)	3 (1.5%)	9 (4.4%)	1 (0.5%)	1 (0.5%)
	**Handicap**					
13	Felt life less satisfying	191 (92.7%)	1 (0.5%)	11 (5.3%)	1 (0.5%)	2 (1.0%)
14	Totally unable to function	193 (93.7%)	2 (1.0%)	11 (5.3%)	0 (0.0%)	0 (0.0%)

### Reliability, reproducibility

For ADD-GOHAI, Cronbach’s alpha was 0.887 and varied from 0.889 to 0.868 when respectively item 12 or 10 was deleted. Item scale correlations varied from 0.41 (item 12) to 0.79 (item10). For ADD-OHIP-14, Cronbach’s alpha was 0.912 and varied from 0.892 to 0.877 when respectively item 2 or 6 was deleted. Item scale correlations varied from 0.48 (item 2) to 0.79 (item 6).

For GOHAI scores, reproducibility was satisfactory with ICC values of 0.919 for the SC score and 0.886 for the ADD score. The ICC values for the GOHAI items were above 0.7 for 4 items (1,8,11,12) and the lowest value (0.551) was obtained for item 6. For OHIP-14 scores, reproducibility was also satisfactory with ICC values of 0.912 for the SC score and 0.863 for the ADD score. The ICC values for the OHIP-14 items were above 0.7 for seven items (3,8,9,11,12,13,14) and the lowest value (0.526) was obtained for item 2.

### Concurrent validity

Lower ADD scores and higher SC scores were significantly associated with perceived poor (or very poor) oral health, perceived poor (or very poor) general health, low level of satisfaction with oral health and with the perception of dental care needs (Table 4).

**Table 4:** Concurrent validity of GOHAI and OHIP-14

		**N**	**GOHAI**		**OHIP-14**	
			**ADD**	**SC**	**ADD**	**SC**
Self-perception of general health	**Good, very good**	**74**	51.92±7.94	2.65±2.67	65.27±6.53	1.61±2.35
	**Moderate**	**79**	46.25±10.97	4.29±3.41	62.10±8.68	2.75±3.27
	**Poor, very poor**	**53**	39.91±11.94	6.21±3.31	57.55±11.42	4.42±3.94
	**-p-value**		**<0.001**	**<0.001**	**<0.001**	**<0.001**
Self-perception of oral health	**Good, very good**	**76**	56.01±3.85	1.30±1.37	68.55±2.27	0.46±.84
	**Moderate**	**65**	47.82±6.12	4.09±2.26	63.38±5.28	2.34±2.11
	**Poor, very poor**	**65**	34.55±9.76	7.68±2.79	53.17±10.44	5.89±3.75
	**-p-value**		**<0.001**	**<0.001**	**<0.001**	**<0.001**
Satisfaction with oral health	**Very satisfied**	**79**	56.16±3.56	1.27±1.30	68.76±1.81	0.39±.72
	**Moderately**	**56**	47.46±5.86	4.25±2.19	63.39±4.38	2.32±1.88
	**Not satisfied**	**71**	35.44±9.91	7.41±2.89	53.58±10.38	5.76±3.71
	**-p-value**		**<0.001**	**<0.001**	**<0.001**	**<0.001**
Self reported need for dental treatment	**Yes**	**128**	41.45±10.65	5.84±3.16	58.61±9.81	4.02±3.57
	**No**	**78**	55.19±5.47	1.50±1.64	67.74±4.17	0.72±1.36
	**-p-value**		**<0.001**	**<0.001**	**<0.001**	**<0.001**

### Discriminant validity

Participants with high levels of oral disease and poor dental status had more frequently low ADD-scores and high SC-scores. Subjects recruited from dispensaries also experienced higher impacts (Table 5). The variable “under dental treatment” was not included in the discriminant analysis due to the small number of participants who answered yes (n=11). The edentulous participants with upper and lower prostheses showed fewer OHRQoL impacts when compared with edentulous patients with no denture or with a single prosthesis (p<0.01). Nevertheless, the variable "wearing a denture" was not considered because the number of FUs outlines more adequately the functional ability of edentulous patients.

**Table 5:** **Discriminant validity of GOHAI and OHIP-14 in univariate and multivariate analysis using 25**^**th**^**and 75**^**th**^**percentile for ADD and SC scores respectively**

		**n**	**GOHAI**		**OHIP-14**	
			**ADD<39.75**	**SC>7**	**ADD<58**	**SC>4**
**Recruitment setting**	Dispensary §	122	41 (33.6%)	42 (34.4%)	39 (32.0%)	32 (26.2%)
	Private	84	10 (11.9%)^*^	10 (11.9%)^*^	16 (19.0%)^+^	15 (17.9%)
	OR_crude_ (95% CI)		3.75 (1.75; 8.00)	3.89 (1.82; 8.30)	2.00 (1.03; 3.88)	1.64 (0.82; 3.26)
	OR_adjusted_ (95% CI)		5.15 (1.87; 14.20)	4.63 (1.78; 12.04)	2.19 (0.97; 4.93)	1.62 (0.72; 3.64)
**Age**	≥ 76 years	50	17 (34.0%)	18 (36.0%)	18 (36.0%)	16 (32.0%)
	65-76 years	156	35 (22.4%)	35 (22.4%)	37 (23.7%)	32 (20.5%)
	OR_crude_ (95% CI)		1.78 (0.89; 3.57)	1.94 (0.97; 3.90)	1.81 (0.91; 3.59)	1.82 (0.90; 3.71)
	OR_adjusted_ (95% CI)		3.03 (1.03; 8.92)	3.05 (1.12; 8.30)	2.89 (1.10; 7.58)	2.89 (1.08; 7.75)
**Gender**	Women	123	35 (28.5%)	35 (28.5%)	37 (30.1%)	30 (24.4%)
	Men	83	16 (19.3%)	17 (20.5%)	18 (21.7%)	17 (20.5%)
	OR_crude_ (95% CI)		1.67 (0.85; 3.26)	1.54 (0.80; 2.99)	1.55 (0.81; 2.97)	1.25 (0.64; 2.46)
	OR_adjusted_ (95% CI)		1.23 (0.53; 2.87)	1.23 (0.54; 2.81)	1.01 (0.47; 2.16)	0.87 (0.40; 1.88)
**Perception of TMJ pain**	Present	34	18 (52.9%)	16 (47.1%)	20 (58.8%)	15 (44.1%)
	Absent	172	33 (19.2%)^*^	36 (20.9%)^§^	35 (20.3%)^*^	32 (18.6%)^§^
	OR_crude_ (95% CI)		4.74 (2.19; 10.27)	3.36 (1.56; 7.23)	5.92 (2.57; 12.17)	3.45 (1.58; 7.52)
	OR_adjusted_ (95% CI)		5.35 (2.04; 14.03)	3.54 (1.40; 8.96)	6.01 (2.51; 14.42)	3.28 (1.38; 7.81)
**Perception of dry mouth**	Present	141	41 (29.1%)	40 (28.4%)	41 (29.1%)	36 (25.5%)
	Absent	65	10 (15.4%)^+^	12 (18.5%)	14 (21.5%)	11 (16.9%)
	OR_crude_ (95% CI)		2.26 (1.05; 4.85)	1.75 (0.85; 3.61)	1.49 (0.75; 2.99)	1.68 (0.79; 3.57)
	OR_adjusted_ (95% CI)		1.63 (0.67; 4.00)	1.39 (0.59; 3.30)	1.11 (0.51; 2.43)	1.40 (0.62; 3.18)
**Number of FUs**	0- 4	90	36 (40.0%)	38 (42.2%)	36 (40.0%)	31 (34.4%)
	5- 8	114	15 (13.2%)^*^	14 (12.3%)^*^	19 (16.7%)^*^	16 (14.0%)^§^
	OR_crude_ (95% CI)		4.40 (2.21; 8.75)	4.99 (2.48; 10.04)	3.33 (1.74; 6.38)	3.22 (1.62; 6.38)
	OR_adjusted_ (95% CI)		2.73 (1.28; 5.82)	2.56 (1.20; 5.60)	2.15 (1.03; 4.51)	2.38 (1.14; 4.95)
**Number of decayed teeth**	7- 28	46	19 (41.3%)	19 (41.3%)	16 (34.8%)	15 (32.6%)
	0- 6	158	31 (19.6%)^#^	32 (20.3%)^#^	38 (24.1%)	31 (19.6%)
	OR_crude_ (95% CI)		2.88 (1.42; 5.84)	2.77 (1.37; 5.60)	1.68 (0.83; 3.42)	1.98 (0.95; 4.12)
	OR_adjusted_ (95% CI)		3.75 (1.39; 10.07)	2.90 (1.13; 7.42)	1.07 (0.44; 2.58)	1.41 (0.59; 3.41)
**Number of teeth**	0- 21	163	47 (28.8%)	47 (28.2%)	47 (28.8%)	41 (25.2%)
	22- 28	43	4 (9.3%)^#^	6 (14.0%)^+^	8 (18.6%)	6 (14.0%)
	OR_crude_ (95% CI)		3.95 (1.34; 11.67)	2.50 (1.01; 6.32)	1.77 (0.76; 4.10)	2.07 (0.82; 5.27)
	OR_adjusted_ (95% CI)		5.78 (1.71; 19.57)	3.32 (1.08; 10.22)	1.90 (0.71; 5.07)	2.46 (0.87; 6.97)

§: The referent categories are in the first row for all the explanatory variables.

* p<0.0001, + p<0.05, # p<0.01, § p<0.001.

Logistic regression models were analysed using the following explanatory variables: age, gender, recruitment setting, perception of TMJ pain, perception of dry mouth, number of teeth (< or ≥ 22), number of dental FUs (≤ or > 4) and number of decayed teeth (< or ≥ 7) (Table 5). Level of education was not included in the multidimensional analysis because a high association was found between the level of education and recruitment setting. Significant associations were found between GOHAI/OHIP scores and age, perception of TMJ pain and number of FUs. Individuals with more than four FUs showed significantly higher ADD and lower SC scores with ORs varying from 2.15 to 2.56. The GOHAI scores were significantly related to the number of teeth, the number of decayed teeth and the recruitment setting. While participants with more than 21 teeth, fewer than seven decayed teeth or those recruited in private practices experienced higher ADD-GOHAI and lower SC-GOHAI scores, no relationship was found for OHIP scores. Gender and perception of dry mouth were not related with the OHRQoL scores.

## Discussion

The purpose of this study was to examine and compare the psychometric properties of two OHRQoL tools among elderly in a developing country (Lebanon) with no generalized public health insurance system. Both GOHAI and OHIP-14 demonstrated good reliability, reproducibility and construct validity for measurement of OHRQoL. The GOHAI and OHIP-14 questionnaires were able to identify participants with impaired oral health, confirmed by a low number of FU and perception of TMJ pain. A strong correlation was found between GOHAI and OHIP-14 but the distribution of the two measures was different with a high prevalence of participants showing no impact with the OHIP-14. Moreover, it appeared that the GOHAI questionnaire was more discriminant and was able to identify participants with a high number of decayed teeth, a low number of teeth or a socially deprived background.

The study has some limitations especially relating to the lack of representativeness of the sample. Patients were recruited in two health care offices in Beirut during a short period. The results thus cannot be applied to elderly living in rural communities or even in other cities in the country. Patients attending those medical structures may also be different from non-attending elderly: they may have higher levels of disease as compared to non-attending people who could be healthier. Inversely, they may have better health insurance coverage or an easier access to medical care than the non-attending population. The level of general health but also of oral diseases might have been under or over estimated. Therefore the results of this survey cannot be used to describe the oral health of the Lebanese elderly population.

It must be noticed that the sample covers an interesting part of the elderly population given that the metropolitan area of Beirut concentrates the highest percentage of Lebanese elderly [1,29]. Moreover, patients with various socio-economic, health and dental status were recruited using two very different medical settings. The characteristics of the patients in the sample were close to the ones of the elderly population living in Beirut [29]**.**

It must also be noticed that potential measurement biases may have occurred given that the same examiner collected clinical and patient-based data. In this study, many elderly people were illiterate and could not complete the questionnaire themselves. The investigator could thus have influenced the answers of the patients during the interview in view of the clinical situation. To avoid this, patients were interviewed before being examined. Generally, the questionnaire delivery modes influence the subjective health status reported by patients, particularly for the psychological aspects of HRQoL [33]. Each approach has its strengths and weaknesses and may be suitable for different circumstances.

The GOHAI and OHIP-14 questionnaires were selected for the study because they are considered to be the instruments of choice for elderly; they are short, allowing higher response rates [7]. The GOHAI and OHIP-14 are similar measures but there are differences in their item content that can affect their ability to detect health-related quality of life outcomes. The present results are in accordance with previous studies which showed that the GOHAI is more successful than OHIP-14 at detecting the oral function problems associated with oral diseases [20-22]. The GOHAI gives greater weight to physical function, pain and discomfort, which are more immediate and common impacts of oral diseases. On the other hand, OHIP-14 explores psychological and social disabilities as well as handicap, which are more severe and less frequent impacts of oral disorders. Thus, the OHIP could play a mediating role in the linkage between OHRQoL and overall well-being in old age [23]. Nevertheless, the high proportion of patients with no impact with OHIP-14 may limit the ability of this questionnaire to detect intra-individual changes in OHRQoL in the elderly and may limit its use in longitudinal studies.

In the present study, GOHAI and OHIP-14 presented different discriminant properties. The GOHAI was more frequently associated with the explanatory variables and was more discriminant than the OHIP-14, as it identified more easily participants with impaired dental status [4]. This aspect is important for researchers planning to conduct a large epidemiological survey among elderly in Lebanon. Many Lebanese elderly cannot afford the cost of dental care and may have high dental treatment needs associated with impaired functional status. In such a situation, health indicators able to identify this part of the population easily, without clinical examination, are of great value.

The number of FUs was used to assess the functional masticatory status of participants. Those with fewer than 4 FUs were considered to have an altered functional status. They experienced greater negative impacts on their oral condition, well-being and function. Additionally, edentulous participants with single dentures experienced lower OHRQoL ADD scores compared with participants who used functional dentures [13]. The number of FUs is an index, which expresses masticatory function and determines masticatory capacity [34,35]. It has been demonstrated that it is a better indicator of masticatory function than the number of teeth present [36]. Furthermore, many studies have established that problems in oral function are more frequent in elderly who have fewer than four FUs; they report difficulties in chewing or swallowing and they tend to avoid hard foods, including meat, vegetables and bread. They can consequently be at risk of malnutrition, which may affect their general health and reduce their life expectancy [30,34-37]. Previous studies have found significant associations between dental status (numbers of teeth, absence of dentures) and nutritional status among the elderly [38,39]. It can thus be hypothesized that unsatisfactory OHRQoL scores associated with a low number of FUs may be a risk factor for malnutrition.

The present study revealed that dental diseases impacted greatly on oral health, well being and functioning of the participants compared with results from other studies conducted in developed countries. The ADD scores were comparable with those reported recently in China and Mexico [15,16]. Additionally, participants recruited from the dispensary (low socio-economic status, illiterate) exhibited higher oral impacts. This is in accordance with previous findings showing that populations living in bad conditions with no social support tend to experience major negative impacts on oral function and well-being [8,40,41].

## Conclusion

The Arabic GOHAI and OHIP14 showed good psychometric properties but the GOHAI identified more easily Lebanese elderly with high dental care needs and impaired oral health. It was more discriminant and better at detecting oral function problems. The results of this study may help to determine the choice of dental indicators for a future national geriatric survey in Lebanon.

## Abbreviations

HRQoL: Health Related Quality of Life; FU: Functional Units; GOHAI: Geriatric Oral Health Assessment Index; ICC: Intra-class Correlation Coefficient; OHIP: Oral Health Impact Profile; OHRQoL: Oral Health Related Quality of Life; OR: Odds Ratio; TMJ: Temporo Mandibular Joint; SC-GOHAI: Simple count Geriatric Oral Health Assessment Index; SC-OHIP: Simple count Oral Health Impact Profile; SC-scores: Simple Count score; SPSS: Statistical Package Software for Social Science; USA: United States of America.

## Competing interests

The authors declare that they have no competing interests.

## Authors’ contributions

NO, SJT, MH, NG contributed with conception and design. NO, LO, NBAN contributed with acquisition of data. NO, SJT, NG contributed with analysis and interpretation of data. NO, SJT, LO involved in drafting the manuscript. SJT, MH, NBAN, NG revised critically the manuscript for important intellectual content. All authors read and approved the final manuscript.
